# Comment on: Antagonistic *in vitro* interaction between olorofim and voriconazole against *Aspergillus fumigatus*

**DOI:** 10.1093/jac/dkag119

**Published:** 2026-03-31

**Authors:** Corinne Pinder, Mike Birch, Jason D Oliver, Derek Law, Daniela Zinzi, John H Rex

**Affiliations:** R&D Department, F2G Ltd, Alderley Park, Macclesfield SK10 4TF, UK; R&D Department, F2G Ltd, Alderley Park, Macclesfield SK10 4TF, UK; R&D Department, F2G Ltd, Alderley Park, Macclesfield SK10 4TF, UK; R&D Department, F2G Ltd, Alderley Park, Macclesfield SK10 4TF, UK; R&D Department, F2G Ltd, Alderley Park, Macclesfield SK10 4TF, UK; R&D Department, F2G Ltd, Alderley Park, Macclesfield SK10 4TF, UK

Dear Editor:

We read with interest the report by Laloum *et al.* regarding their data showing an antagonistic interaction between olorofim and voriconazole against *Aspergillus fumigatus in vitro*.^1^ We would like to extend their report by connecting it with other data that were in press at *JAC* the time of review of their paper. We concur that *in vitro* antagonism can be shown between olorofim and voriconazole against many, but not all, isolates of *A. fumigatus*. We find their agar-based demonstrations showing that the interaction is focused within a narrow band around the MIC of the azole to be especially instructive.

As called for by Laloum *et al*., we have extended their observations on voriconazole by publishing data examining the *in vitro* combination of olorofim with posaconazole, isavuconazole, fluconazole as well as amphotericin B, terbinafine and caspofungin against *A. fumigatus* and non-*fumigatus* species.^2^ For all isolates of *A. fumigatus*, *A. flavus*, *A. terreus* and *A. niger* we observed unidirectional antagonism of olorofim activity by isavuconazole and posaconazole whereas fluconazole had no interaction with olorofim. Similar to Laloum *et al.*, we found that voriconazole antagonized olorofim activity for some, but not all of the isolates tested (62.5%^2^ and 48%^1^ of isolates). Similarly, van Rhijn *et al.*^3^ also observed different interactions between olorofim and voriconazole dependent on the *Aspergillus* isolate tested (antagonism for a wild-type isolate and indifference for an azole-resistant isolate) that was mistakenly reported as uniform antagonism in the original publication and then quoted as ‘strong antagonism’ for both isolates by Laloum *et al*. Taken together, there is now evidence that mould-active azoles antagonize olorofim *in vitro* against *Aspergillus* spp. but this phenomenon is not consistent across isolates/species for voriconazole and varies further across analytical methods. Further highlighting the method-, genus-, species- and isolate-dependent nature of these findings, indifference has been observed against *Madurella mycetomatis*^4^ and *Scedosporium aurantiacum*.^5^

Regarding the nature of the antagonistic interaction between azoles and olorofim, we note that Laloum *et al.*^1^ also saw that the effect was seen at concentrations of the azole at or just below the azole MIC,^2^ probably as a result of this condition causing upregulation of the pyrimidine biosynthetic pathway.^3^ However, as with most of our experiments,^2^ resulting raised olorofim MICs remained within the wild-type distribution for olorofim-susceptible *Aspergillus* spp.^1,6^

Some of these *in vitro* findings have been validated *in vivo*, addressing one of Laloum and colleagues’ call to action. Using a murine model of invasive pulmonary aspergillosis, Darlow *et al.* reported that antagonism could be shown between *A. fumigatus* and the mould-active azole posaconazole.^7^ These authors conclude that a combination of olorofim and a mould-active azole is probably efficacious in wild-type infections but may be suboptimal in azole-resistant infections where, although there would be minimal contribution of the azole to antifungal activity, it could antagonize olorofim to produce a submaximal effect.

Importantly, preclinical antagonism has been noted between currently approved antifungal agents but has not precluded combination therapy.^8^ Laloum *et al*. called for the investigation of the efficacy of combination treatment in clinical settings and we have done this by examining data from our recently completed open-label, single-arm, Phase 2b study (NCT03583164, Study 32) of patients with few or no treatment options for proven IFD or probable invasive pulmonary aspergillosis based on the 2008 European Organization for Research and Treatment of Cancer/Mycoses Study Group (EORTC/MSG) definitions.^9^ We previously analysed this for the first 100 enrolled patients and did not find evidence of a clinical consequence in those who received an azole in combination with olorofim.^10^ The data from the full 203-patient dataset have now been analysed and reveal no consistent differences in outcome or safety risk when olorofim is used as monotherapy compared with its use in combination therapy with either a mould-active azole or with fluconazole; a full manuscript has been submitted to *JAC* alongside this letter.^11^

In conclusion, we thank Laloum and colleagues for confirming our preclinical findings with *Aspergillus* and for providing instructive visual demonstrations of the concentration-dependent unidirectional antagonism of olorofim by the mould-active azoles. Importantly, antagonism is not seen with fluconazole, amphotericin B, terbinafine, or caspofungin. Finally, no evidence of clinical consequence of these findings has been seen in analyses of data available to date.

## References

[1] Laloum I, Bidaud AL, Recalt E et al Antagonistic *in vitro* interaction between olorofim and voriconazole against *Aspergillus fumigatus*. J Antimicrob Chemother 2025; 80: 3452–61. 10.1093/jac/dkaf393 41140055

[2] Pinder C, Lebedinec R, Oliver JD et al *In vitro* evaluation of olorofim and antifungal combinations against *Aspergillus* and *Candida* species. J Antimicrob Chemother 2025; 80: 3139–49. 10.1093/jac/dkaf354 41025311 PMC12596045

[3] van Rhijn N, Hemmings S, Storer ISR et al Antagonism of the azoles to olorofim and cross-resistance are governed by linked transcriptional networks in *Aspergillus fumigatus*. mBio 2022; 13: e0221522. 10.1128/mbio.02215-22 36286521 PMC9765627

[4] Lim W, Eadie K, Konings M et al *Madurella mycetomatis*, the main causative agent of eumycetoma, is highly susceptible to olorofim. J Antimicrob Chemother 2020; 75: 936–41. 10.1093/jac/dkz529 31904836 PMC7069493

[5] Rollin-Pinheiro R, Xisto MIDDS, de Castro-Almeida Y et al Pandemic Response Box(R) library as a source of antifungal drugs against *Scedosporium* and *Lomentospora* species. PLoS ONE 2023; 18: e0280964. 10.1371/journal.pone.0280964 36735743 PMC9897528

[6] Astvad KMT, Jørgensen KM, Hare RK et al Olorofim susceptibility testing of 1,423 Danish mold isolates obtained in 2018–2019 confirms uniform and broad-spectrum activity. Antimicrob Agents Chemother 2020; 65: 01527–0. 10.1128/AAC.01527-20 PMC7927864 33020160

[7] Darlow CA, Farrington N, Martson A-G et al Antagonistic interaction between posaconazole and olorofim in a murine model of invasive pulmonary aspergillosis. J Antimicrob Chemother 2025; 80: 3150–9. 10.1093/jac/dkaf355 40990169 PMC12596047

[8] Johnson MD, Macdougall C, Ostrosky-Zeichner L et al Combination antifungal therapy. Antimicrob Agents Chemother 2004; 48: 693–715. 10.1128/AAC.48.3.693-715.2004 14982754 PMC353116

[9] Maertens JA, Thompson GR, Spec A et al Olorofim for the treatment of invasive fungal diseases in patients with few or no therapeutic options: a single-arm, open-label, phase 2b study. Lancet Infect Dis 2025; 25: 1177–88. 10.1016/S1473-3099(25)00224-5 40541222

[10] Zinzi D, Birch M, Harvey E et al Outcomes of invasive fungal disease in a subgroup of patients receiving olorofim/azole combination in an open label salvage study. Eleventh Trends in Medical Mycology, Athens, Greece, 2023. Abstract P412.

[11] Maertens J, Chen S, Thompson GR et al Olorofim in combination with azole antifungals: results from the phase 2 salvage therapy study. J Antimicrob Chemother 2026. 10.1093/jac/dkag118 PMC13036598 41914555

